# Multimodality approach to the no-entry ventricle for VT ablation: first European experience of the RA-to-LV percutaneous access—a case report

**DOI:** 10.1093/ehjcr/ytag384

**Published:** 2026-06-16

**Authors:** Javier Sanaú, Julian Rodriguez García, Josep Comín-Colet, Ignasi Anguera, Paolo Domenico Dallaglio

**Affiliations:** Arrhythmia Unit, Department of Cardiology, Hospital Universitari de Bellvitge, L’Hospitalet de Llobregat, Carrer Feixa Llarga s/n, Barcelona 08907, Spain; Bioheart-Cardiovascular Diseases Group, Institut D’Investigació Biomèdica de Bellvitge–IDIBELL, L’Hospitalet de Llobregat, Avda. Gran Vía de l'Hospitalet 199, Barcelona 08908, Spain; Arrhythmia Unit, Department of Cardiology, Hospital Universitari de Bellvitge, L’Hospitalet de Llobregat, Carrer Feixa Llarga s/n, Barcelona 08907, Spain; Bioheart-Cardiovascular Diseases Group, Institut D’Investigació Biomèdica de Bellvitge–IDIBELL, L’Hospitalet de Llobregat, Avda. Gran Vía de l'Hospitalet 199, Barcelona 08908, Spain; Arrhythmia Unit, Department of Cardiology, Hospital Universitari de Bellvitge, L’Hospitalet de Llobregat, Carrer Feixa Llarga s/n, Barcelona 08907, Spain; Bioheart-Cardiovascular Diseases Group, Institut D’Investigació Biomèdica de Bellvitge–IDIBELL, L’Hospitalet de Llobregat, Avda. Gran Vía de l'Hospitalet 199, Barcelona 08908, Spain; University of Barcelona; Innovation, Research and Universities Department, Gerència Territorial Metropolitana Sud, Institut Català de la Salut; CIBER-CV, Gran Via de les Corts Catalanes, 585, Barcelona 08007, Spain; Arrhythmia Unit, Department of Cardiology, Hospital Universitari de Bellvitge, L’Hospitalet de Llobregat, Carrer Feixa Llarga s/n, Barcelona 08907, Spain; Bioheart-Cardiovascular Diseases Group, Institut D’Investigació Biomèdica de Bellvitge–IDIBELL, L’Hospitalet de Llobregat, Avda. Gran Vía de l'Hospitalet 199, Barcelona 08908, Spain; Arrhythmia Unit, Department of Cardiology, Hospital Universitari de Bellvitge, L’Hospitalet de Llobregat, Carrer Feixa Llarga s/n, Barcelona 08907, Spain; Bioheart-Cardiovascular Diseases Group, Institut D’Investigació Biomèdica de Bellvitge–IDIBELL, L’Hospitalet de Llobregat, Avda. Gran Vía de l'Hospitalet 199, Barcelona 08908, Spain

**Keywords:** Case report, Ventricular tachycardia ablation, Mechanical valves, AV septum, Multimodality imaging

## Abstract

**Background:**

Catheter ablation of ventricular tachycardia (VT) in patients with both mechanical aortic and mitral valves remains challenging, as conventional retrograde or transseptal approaches are not feasible due to the risk of damaging prostheses. Alternative strategies such as thoracotomy, epicardial access, or stereotactic radiotherapy may be unsuitable due to anatomical or technical constraints. The right atrium-to-left ventricle (RA-to-LV) puncture through the inferoseptal process has recently emerged as a potential solution, yet real-world experience remains limited.

**Case summary:**

An 81-year-old man with rheumatic heart disease, double mechanical left-sided valve replacement, chronic kidney disease, and recurrent drug-refractory VT was referred for catheter ablation. Multimodal imaging using cardiac magnetic resonance and contrast-enhanced CT processed with ADAS3D software enabled identification of the arrhythmogenic substrate in the basal lateral LV and preprocedural planning of a safe puncture trajectory. Under general anaesthesia and uninterrupted anticoagulation, a percutaneous RA-to-LV puncture was performed with real-time guidance from electroanatomical mapping, CT-fluoroscopy integration, and intracardiac echocardiography. High-density mapping revealed a basal lateral scar with areas of conduction deceleration. Targeted ablation eliminated abnormal potentials and rendered VT non-inducible. A small restrictive iatrogenic Gerbode defect was observed without haemodynamic impact at 4-month follow-up echocardiogram. The patient recovered uneventfully and remained free of VT and ICD therapies at 5-month follow-up.

**Discussion:**

This case suggests that RA-to-LV access is feasible and safe in a highly selected patient when thorough multimodal planning is undertaken. This report represents the first European experience and supports the potential role of this technique in patients with no-entry ventricle.

Learning points

- RA-to-LV puncture through the inferoseptal process may enable LV access for VT ablation in a ‘no entry’ ventricle.
- Integration of CT/MRI reconstructions with ADAS3D may improve procedural safety and facilitate the learning curve.

## Introduction

Catheter ablation of ventricular tachycardia (VT) in patients with double left-sided mechanical valves remains a significant challenge, as the retrograde *trans*-aortic or antegrade transseptal atrial approaches are unsuitable because of the potential physical interaction between the ablation system and prostheses in both the aortic and mitral positions.^1,2^

## Summary figure

**Figure:**
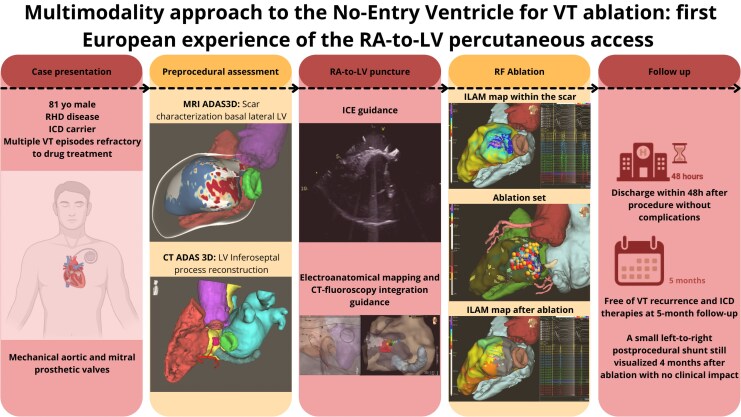


## Case presentation

An 81-year-old male with history of hypertension, diabetes mellitus, chronic kidney disease stage 4, and persistent atrial fibrillation. He had a history of rheumatic heart disease (RHD) requiring mitral and aortic valve replacement with mechanical prostheses, and a defibrillator (ICD) implantation in secondary prevention for VT. During the last year he presented several episodes of slow VT (cycle lengths between 410 and 460 ms) requiring multiple ICD therapies, ICD reprogramming, recurrent hospital admissions for heart failure, and progressive escalation of antiarrhythmic drugs. Amiodarone had been withdrawn due to inefficacy and intolerance and a scheme with sotalol plus mexiletine was started but proved to be ineffective in avoiding new VT episodes. Cardiac magnetic resonance (CMR) showed mildly reduced ejection fraction (43%), increased LV indexed volume (103 ml/m2), and transmural late gadolinium enhancement (LGE) in the basal lateral segment which may have been caused by an embolic event in absence of coronary artery disease. Two VT morphologies suggesting an origin in the LV had been documented (*Figure 1*).

**Figure 1:**
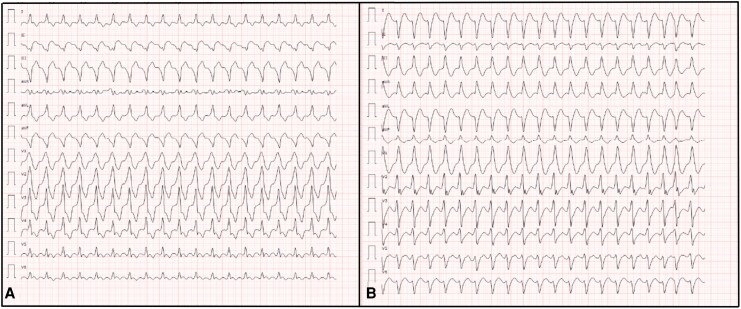
Electrocardiograms during ventricular tachycardia. *(A)* The electrocardiogram of VT1 shows positive electrical concordance in precordial leads and superior left axis. *(B)* The electrocardiogram of VT2 shows RBBB morphology with rS in V2 and transition in V3, with DII/DIII discordance and negative DI and aVL leads.

Due to refractoriness to multiple drug treatment, VT ablation was considered. Based on these findings, a percutaneous right atrium-to-left ventricule (RA-to-LV) puncture through the inferoseptal process (ISP) was planned to obtain endocardial access to the LV for VT ablation.

Previously performed CMR was reconstructed using ADAS 3D software. This software enables semiautomatic postprocessing combining machine learning and operator input to delineate endocardial and epicardial contours. After segmentation of LV walls, a 3D reconstruction is generated with layered wall analysis and tissue characterization based on LGE signal intensity. This analysis revealed an area of border zone tissue possibly hosting slow conducting channels in the endocardial layers of the scar in the basal lateral segment (*Figure 2A*).

**Figure 2:**
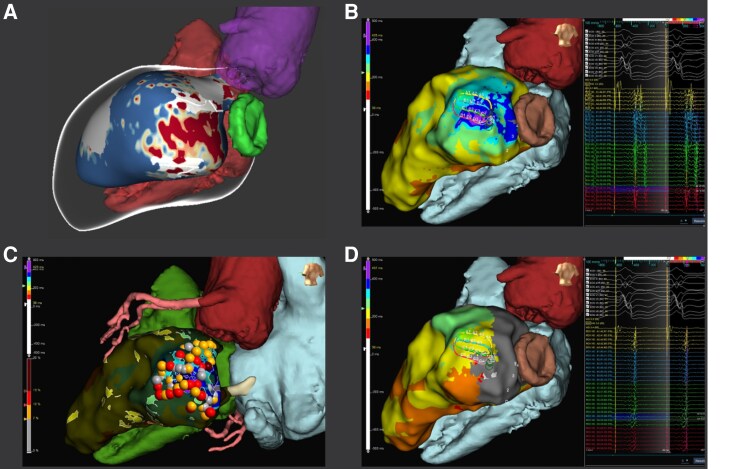
*(A)* CMR with ADAS3D reconstruction showing the LV and adjacent structures. The mechanical mitral prosthesis (green), aorta (purple), and RV (red) are displayed for spatial reference. A dense scar in the basal lateral LV is visualized (red) with a surrounding border-zone (white-orange), within which distinct conduction channels are identified (white lines). *(B)* ILAM map demonstrating areas of slow conduction within the basal lateral scar, matching the channels identified on CMR. Mechanical mitral prosthesis (brown), aorta (red), and RV (light blue) are displayed. *(C)* Ablation set in the lateral basal scar. *(D)* Post-ablation ILAM showing complete elimination of late potentials within the targeted region.

As part of the preoperative evaluation, an electrocardiogram-gated contrast-enhanced CT scan was performed. Images reconstructed at mid-diastole were processed for analysis using ADAS 3D software. First, the optimal path from the RA-to-LV was estimated by using multiplanar reconstruction images and a 3D path function (*Figure 3*). As the path needed to penetrate the ISP, it was carefully planned to avoid coronary arteries within this region, including the distal right coronary artery and the atrioventricular nodal artery. The initial pathway of the atrioventricular nodal artery could be detected and depicted to avoid it during the puncture. Then, the area of the puncture was transformed into a virtual target. Important landmark structures, including the coronary arteries and coronary sinus, were additionally reconstructed in a volume-rendered image with extracardiac and intracardiac radiopaque structures, such as mitral and aortic mechanical prostheses, and ICD lead. This allowed anatomical integration of CT imaging with fluoroscopy.

**Figure 3:**
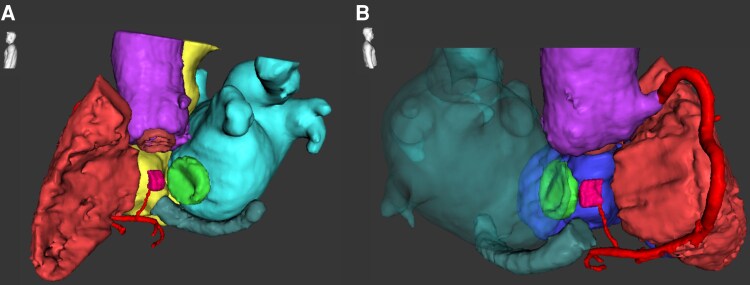
Multiplanar ADAS 3D reconstruction showing the target puncture site within the inferoseptal process of the LV (pink). Surrounding cardiac and prosthetic structures are displayed to illustrate the anatomical references: RV (red), aorta (purple), coronary sinus (grey), mechanical aortic prosthesis (brown), mechanical mitral prosthesis (green), right coronary artery and AV node artery (red), and LA (blue). *(A)* Left lateral view. *(B)* Right lateral view.

The procedure was performed under general anaesthesia. Acenocoumarol was uninterrupted (INR 2.5 at procedure), and intraprocedural heparin was administered to maintain ACT >300 s. The procedure was guided by the CT reconstruction on fluoroscopy with real-time assistance of the electroanatomical mapping system (EnSite X; Abbott) and intracardiac echocardiography (ICE, Soundstar Ultrasound Catheter; Biosense Webster Diamond Bar).

A deflectable long sheath (Agilis; Abbott) was advanced towards the inferomedial aspect of the RA adjacent to the inferior portion of the ISP. A guiding catheter sheath (MultiPurpose; Balton) offered better stability and contact with the RA wall. Once adequate contact was achieved under ICE visualization (*Figure 4*, Supplementary material online, *Video S1*), a 0.035 in straight tip guidewire (Emerald; Cordis), electrically isolated using a noncompliant angioplasty balloon (8 mm diameter × 40 mm length—Armada, BioSud Argentina), was connected to a standard electrocautery system and RF energy was delivered. The precise site of puncture was chosen according to the real-time CT integration on fluoroscopy, ICE, and the magnetically guided wire. During RF delivery across the ISP, the guidewire and the balloon were advanced to the LV chamber, perforating the ISP under direct ICE and fluoroscopy guidance (*Figure 5*, Supplementary material online, *Video S1*). This RF delivery induced VF, which immediately organized into a VT (same morphology of the clinical VT1) requiring external cardioversion. Upon gaining access into the LV, the tract was dilated to allow sheath entry into the LV (Supplementary material online, *Videos S2* and *S3*).

**Figure 4:**
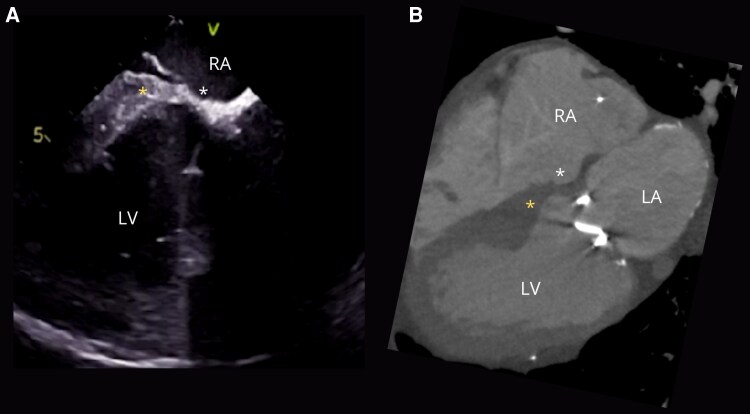
*(A)* Intracardiac echocardiography (ICE) view used to guide the puncture trajectory, in which white asterisk marks the ISP and the yellow asterisk marks the inferoseptal LV. *(B)* Cardiac CT replication of the sectional plane of the ICE view. RA, right atrium; LA, left atrium; LV, left ventricle.

**Figure 5:**
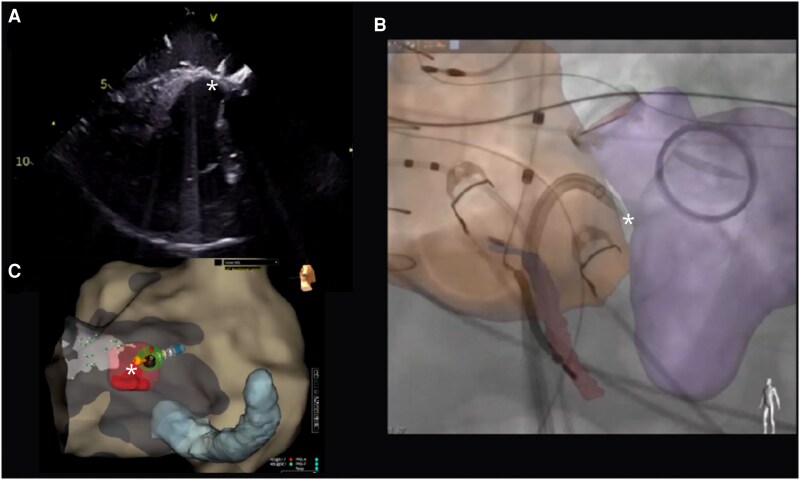
The puncture was guided using real-time ICE *(A)*, fluoroscopy *(B)*, and electroanatomical mapping *(C)*. The white asterisk marks the ISP. ADAS3D integration enabled precise localization of the target region (red) within the ISP and its alignment with the electroanatomical map, facilitating a safe puncture trajectory.

High-density electroanatomical mapping was performed during RV apex stimulation to have a homogeneous myocardial activation. Voltage mapping revealed a lateral basal scar (*Figure 2B*). Isochronal late activation mapping (ILAM) showed late potentials with areas of deceleration (*Figure 2C*). While mapping, VT2 was induced, identifying a critical isthmus within the scar where isochronal crowding had been identified. After catheter ablation targeting these abnormal potentials and the VT isthmus, ILAM showed absence of previously found isochronal crowding and elimination of late potentials (*Figure 2D*). No sustained VT could be induced postablation. When the ablation catheter and the deflectable sheath were withdrawn to the RA chamber, a residual iatrogenic Gerbode defect was visualized with ICE showing a LV to RA high speed restrictive flow. No pericardial effusion was seen with ICE.

The patient had an uneventful recovery. No new arrhythmias or conduction disturbances occurred. His ICD was reprogrammed, and no further appropriate shocks or VT episodes were noted during the index hospitalization. The patient was discharged on Day 2 with optimized heart failure therapy and continuation of anticoagulation. At 5-month follow-up, the patient remains free of VT recurrence and ICD therapies. A transthoracic echocardiogram was performed after 4 months and a small residual Gerbode defect with restrictive flow (peak velocity of 4.2 m/s) and a Qp/Qs ratio of 1.3 could still be visualized.

## Discussion

In this case, access through minithoracotomy or an epicardial approach was not considered because of potential post-surgical adhesions in the pericardial space and potential complications due to withdrawal of anticoagulation. Stereotactic radioablation therapy was not used either due to limitations in the absence of electroanatomical mapping. Therefore, the RA-to-LV puncture was selected.^3^ This approach, first described by Santangeli *et al.*, and later supported by a multicentre registry,^4–8^ comprises a percutaneous puncture from RA through the ISP, creating a Gerbode-like tract that enables catheter access to the LV for endocardial VT ablation.

This case suggests feasibility and short-term safety of percutaneous *trans*-right atrial puncture to the ISP in a highly selected patient. Importantly, this procedure was successfully performed in an elderly patient with double left-sided mechanical valves and substantial comorbidity.

A key strength of our approach was the thorough preprocedural reconstruction of the puncture site with ADAS3D to integrate the CT reconstruction with real-time fluoroscopy and electroanatomical mapping. This approach may increase safety and reduce the learning curve.

To our knowledge, this report represents the first successful application of this technique in Europe. Although RHD is less common in Europe than in low- or middle-income countries, a recent increase in its incidence rate has been reported.^9^ Since surgical valve replacement remains a cornerstone of care for RHD, this may increase the likelihood of encountering patients with double left-sided mechanical valves requiring future VT ablation. In such complex anatomies, the RA-to-LV approach is emerging as a valuable alternative by providing safe LV access while preserving prosthetic integrity. Ongoing refinement of puncture tools, delivery systems, and intracardiac imaging may further enhance procedural safety and efficiency.

Despite the favourable outcome, several limitations must be acknowledged. A single highly selected case is described, follow-up duration is short, and long-term durability of both VT suppression and the residual iatrogenic Gerbode-like defect remains to be established. Furthermore, the RA-to-LV puncture technique requires advanced expertise in structural interventions, ICE, and electroanatomical mapping. The learning curve may be significant, and procedural safety is likely dependent on thorough preprocedural planning and operator experience.^10^

Although spontaneous closure of the left-to-right postprocedural shunt has been reported,^7^ in our patient it was still visualized 4 months after the ablation. Considering its small size and its restrictive flow, the haemodynamic consequences are expected to be minimal. No right atrial or ventricular enlargement was detected at echocardiography. Quantification of the Qp/Qs ratio has been performed with transthoracic echocardiography resulting in a value of 1.3. This measurement may be inaccurate due to the method used and should be repeated with CMR. However, the patient has had no symptoms of heart failure or worsening of his functional class since the ablation. Thus, in absence of clinical need, a CMR has not been programmed. Transthoracic echocardiography has been planned every 12 months within the first 3 years of follow-up. Theoretically, if closure of the AV defect were deemed necessary for any reason, this could be accomplished with percutaneous occluders.

In summary, this case suggests that the RA-to-LV ISP puncture technique is reproducible in a highly selected patient with a no-entry ventricle for VT ablation, when performed in centres with extensive experience in complex VT ablation and structural cardiac interventions. It highlights the value of innovation and multidisciplinary collaboration in the management of complex arrhythmogenic substrates.

Written informed consent was obtained from the patient for the publication of this case report and accompanying images, in compliance with COPE guidelines. The data underlying this article will be shared on reasonable request to the corresponding author.

## Supplementary Material

ytag384_Supplementary_Data

## Data Availability

The data underlying this article will be shared on reasonable request to the corresponding author.

## Lead author biography

Paolo Domenico Dallaglio, MD, PhD, is a consultant cardiologist specialized in cardiac electrophysiology with over 15 years of experience in the management of complex arrhythmias. He is accredited by the European Heart Rhythm Association and holds a postgraduate degree in Health Sciences Statistics. He earned his PhD cum laude from the University of Barcelona. He currently serves as senior consultant at Bellvitge University Hospital, focusing on high-complexity ablation procedures and multimodal imaging integration. His research interests include ventricular arrhythmias, advanced ablation technologies, and cardiac imaging, with more than 40 peer-reviewed publications.
